# Does the Time of Day Play a Role in the Acute Effect of *p*-Synephrine on Fat Oxidation Rate during Exercise in Women? A Randomized, Crossover and Double-Blind Study

**DOI:** 10.3390/nu14235030

**Published:** 2022-11-26

**Authors:** Jorge Gutiérrez-Hellín, Juan Del Coso, Millán Aguilar-Navarro, David Varillas-Delgado, Carlos Ruiz-Moreno, Álvaro López-Samanés, Francisco J. Amaro-Gahete, Alejandro Muñoz

**Affiliations:** 1 Exercise and Sport Science, Faculty of Health Sciences, Universidad Francisco de Vitoria, 28223 Pozuelo, Spain; 2 Centre for Sport Studies, Rey Juan Carlos University, 28943 Fuenlabrada, Spain; 3 Exercise Physiology Laboratory, Camilo José Cela University, 28692 Villanueva de la Cañada, Spain; 4 School of Physiotherapy, Faculty of Health Sciences, Universidad Francisco de Vitoria, 28223 Pozuelo, Spain; 5 Department of Medical Physiology, School of Medicine, University of Granada, 18071 Granada, Spain

**Keywords:** phytochemical, sports nutrition, circadian rhythm, carbohydrate oxidation, female athlete

## Abstract

*p*-Synephrine is deemed a safe and effective substance to increase fat utilization during exercise of low-to-moderate intensity in men but not in women. Additionally, the existence of a diurnal variation in substrate utilization has been documented during exercise with enhanced fat oxidation in the evening compared with early morning. However, it remains unknown whether there is an interaction between the effect of *p*-synephrine and the time of the day on fat oxidation during exercise. This study aimed to evaluate the effect of the acute ingestion of 3 milligram of *p*-synephrine per kilogram of body mass (mg/kg) on fat oxidation during exercise of increasing intensity when the exercise is performed in the morning vs. the evening. Using a randomized, double-blind, placebo-controlled experimental design, 16 healthy and active women performed four identical exercise trials after the ingestion of 3 mg/kg of *p*-synephrine and 3 mg/kg of a placebo (cellulose) both in the morning (8–10 am) and in the evening (5–7 pm). In the exercise trials, the substances were ingested 60 min before an incremental test on a cycle ergometer with 3 min stages at workloads from 30 to 80% of maximal oxygen uptake (VO_2_max). Substrate oxidation rates were measured by indirect calorimetry. In each trial, the maximum rate of fat oxidation (MFO) and the intensity that elicited MFO (Fatmax) were measured. A two-way analysis of variance (time-of-the day × substance) was used to detect differences among the trials. With the placebo, MFO was 0.25 ± 0.11 g/min in the morning and 0.24 ± 0.07 g/min in the evening. With *p*-synephrine, MFO was 0.26 ± 0.09 g/min in the morning and 0.21 ± 0.07 g/min in the evening. There was no main effect of substance (*p* = 0.349), time of day (*p* = 0.186) and the substance × time of day (*p* = 0.365) on MFO. Additionally, Fatmax was reached at a similar exercise intensity with the placebo (41.33 ± 8.34% VO_2_max in the morning and 44.38 ± 7.37% VO_2_max in the evening) and with *p*-synephrine (43.33 ± 7.24% VO_2_max in the morning and 45.00 ± 7.43% VO_2_max in the evening), irrespective of the time of day with no main effect of substance (*p* = 0.633), time of day (*p* = 0.191), or interaction (*p* = 0.580). In summary, the acute intake of 3 mg/kg of *p*-synephrine before exercise did not increase MFO and Fatmax, independently of the time of day, in female athletes. This indicates that the time of day is not a factor explaining the lack of effectiveness of this substance to enhance fat oxidation during aerobic exercise in women.

## 1. Introduction

*p*-Synephrine is a phytochemical naturally present in *Citrus aurantium* [1,2]. This phytochemical is also present in fruits and leaves of other *Citrus* species [3], such as Seville Orange, Sour Orange, Green Orange, Zhi Shi, and Kijitsu, although its concentration greatly varies among species [4]. Currently, the popularity of *p*-synephrine is growing worldwide within the market of dietary supplements, especially among those exercise enthusiasts that train to obtain body weight reductions [5]. Although, initially, *p*-synephrine was deemed as a performing-enhancing substance, this substance has demonstrated a null effect to increase sports performance to date [6]. The popularity of *p*-synephrine is linked to recent literature pointing toward the benefit of acute *p*-synephrine intake to enhance lipid utilization during exercise [7,8,9,10]. Collectively, these investigations have found that the ingestion of between 2 and 3 milligrams of pure p-synephrine per kilogram of body mass ~60 min before exercise increases the rate of fat oxidation during exercise of low-to-moderate intensity [7,8,9,10]. Additionally, it has been found that *p*-synephrine intake increases the total amount of fat oxidized during an acute bout of aerobic exercise (i.e., 60 min of cycling at moderate intensity) [11]. All these investigations have been carried out in samples of athletes or active individuals with a predominance of men, while a recent investigation in women has disputed the effect of *p*-synephrine to enhance fat oxidation during exercise [12]. Only in women has an increase in body temperature with *p*-synephrine been reported that may affect its potential benefits on fat oxidation during exercise [12].

All the above studies were conducted in a stable morning environment [7,8,9,10,11,12]. Previous investigations on circadian rhythms showed that there is a diurnal variation in the rate of fat utilization during exercise that may interfere with the effectiveness of *p*-synephrine to enhance fat oxidation in women. In these investigations, maximal fat oxidation during aerobic exercise was increased by ~10% in the evening compared with the morning [13,14,15]. Again, this fluctuation in the use of fat during exercise is gender specific, as a recent study has reported that fat oxidation is similar in the morning and the evening in women [16]. Lastly, a recent investigation studied the synergistic effect of acute caffeine intake and time of day (morning vs. evening) on fat oxidation rates during exercise [15]. In that investigation, there was a higher fat oxidation rate in the evening while the acute ingestion of caffeine increased fat utilization during exercise independent of the time of day. This investigation suggests that there is a more optimal synergistic effect of combining a substance that increases fat oxidation (e.g., caffeine) with the selection of time of day to favor fat utilization within skeletal muscle. However, a similar experiment has not been carried out on *p*-synephrine, and it remains unknown whether there is an interaction between the effect of *p*-synephrine and the time of the day on fat oxidation during exercise. Additionally, the lack of effect of *p*-synephrine to improve fat oxidation in women may be because this effect has only been tested in the morning in women [12]. This study aimed to evaluate the effect of the acute ingestion of 3 milligrams of *p*-synephrine per kilogram of body mass (mg/kg) on fat oxidation during exercise of increasing intensity when the exercise is performed in the morning vs. the evening.

## 2. Materials and Methods

### 2.1. Participants

Sixteen women, categorized as physically active, voluntarily participated in this investigation. Inclusion criteria were: (i) being non-smokers, (ii) having no previous history of cardiopulmonary diseases, (iii) having not suffered musculoskeletal injuries in the previous 6 months, (iv) having regular duration of their menstrual cycle for the previous 6 months and (v) not suffering any type of menstrual disorders such as dysmenorrhea, amenorrhea, or strong symptoms associated with pre-menstrual syndrome. Exclusion criteria were: (i) >45 years of age, (ii) sedentarism, and (iii) the use of medicaments or oral contraceptives. This information was obtained from a pre-participation screening that included a medical and training history. Participants signed an informed written consent form to participate in the investigation. The study was approved by the Francisco de Vitoria University Research Ethics Committee (UFV 18-2020) and followed the principles of Declaration of Helsinki 1964 (last update 2013).

### 2.2. Experimental Design

A randomized, double-blind and placebo-controlled design was used, with each participant serving as her control. Each participant performed four identical experimental sessions completing the same test protocol but under four different conditions: (i) morning (8:00–10:00) and ingestion of 3 mg/kg of *p*-synephrine (99.2% purity; synephrine HCl, Liftmode, USA), (ii) morning (8:00–10:00) with ingestion of 3 mg/kg of a placebo substance (Cellulose, Guinama, Spain), (iii) evening (17:00–19:00) with 3 mg/kg of *p*-synephrine and (iv) evening (17.00–19:00) with 3 mg/kg of placebo. An interval period of >48 h between experimental sessions was used to allow for sufficient recovery from exercise testing sessions. Substances were administered in an opaque and unidentifiable capsule and ingested with 150 mL of tap water 60 min before the onset of the experimental trials. The assignment of the trial order was performed by an independent investigator using a randomizer program (www.randomizer.org, accessed on 9 January 2021). To ensure standardization of the measurements, all tests were completed at the same lab, using the same testing devices, and handled by the same researchers. All participants performed the experimental tests in the mid-luteal phase of their menstrual cycle.

### 2.3. Pre-Experimental Procedure

One week before the first trial, participants were weighed and morphologically analyzed by bioimpedance (RD-901BK36, Tanita, Tokyo, Japan). Once the bioimpedance measurement was finished, the participants filled a morningness–evening questionnaire for detecting individual chronotype [17]. Afterward, they underwent a standardized warm-up that included 10 min at 50 Watts (W) on a cycle ergometer (Ergoline, Ergoselect 4, Bitz, Germany). Then, a ramp exercise test on the cycle ergometer was completed with successive 15 W load increments every 1 min until volitional fatigue to determine maximal oxygen uptake (VO_2_max). During the test, participants chose a cadence of 70 to 90 rpm. Oxygen uptake (VO_2_) was measured during the whole test using a breath-by-breath analyzer (Ergostik-Geratherm Respiratory, Bad Kissingen, Germany). The maximum value of VO_2_ was deemed maximum when participants reached at least three of the following criteria: (i) VO_2_ stabilization despite increases in ergometric power, (ii) a respiratory exchange ratio higher than 1.10, (iii) a rating of perceived exertion larger than 19 points (6–20 point Borg scale), (iv) heart rate higher than the 90% of maximal heart rate age-predicted estimation (HRmax = 220-age) [18] and (v) unable to maintain a cadence >50 rpm in the last load. The data obtained during this test were used to calculate the workload for the experimental trials (as% of VO_2_max), by using an individual regression equation for the relationship workload–VO_2_.

### 2.4. Experimental Trials

Before each experimental trial, participants confirmed that they had refrained from strenuous exercise the day before. Participants were also required to avoid alcohol, caffeine, and other stimulants 24 h before each trial. They were instructed to complete a 24 h dietary record on the day before the first trial and to follow the same dietary pattern during all visits. The diet was identical 24 h before each experimental trial, irrespective of the time of the trial (i.e., morning or evening). During experimental trials, participants arrived at the laboratory in a fasted state (at least 8 h after their last meal) and two hours after ingesting 7 mL/kg of water. Upon arrival, urine specific gravity was measured with a refractometer (MASTER-S28M, Atago, Japan); urine specific gravity levels <1020 were established to ensure a euhydration status. Subsequently, the participants ingested the assigned capsule with the treatment (i.e., p-synephrine or placebo) and rested in a supine position for 60 min. During the last minutes of the resting period, heart rate (Wearlink, Polar, Finland) and systolic/diastolic blood pressure (M6 Comfort, Omron, Kyoto, Japan) were measured while the participants rested on a stretcher. After this, the tympanic temperature was measured in triplicate by using an infrared tympanic thermometer (Thermoscan 7, Braun, Kronberg, Germany; [19]). Thereafter, participants voided, and a urine sample was obtained to measure pre-exercise urine concentrations of *p*-synephrine and 4-hydroxymandelic acid, as previously described [12].

After resting measurements, participants performed a warm-up consisting of 10 min at 30% VO_2_max. Afterward, participants started the exercise test, which consisted of 10% VO_2_max increments every 3 min until the respiratory exchange ratio (RER) >1.00 [20], starting at 30% VO_2_max. During exercise, expired gases were collected with a highly reliable stationary breath-by-breath device (Ergostik-Geratherm Respiratory, Bad Kissingen, Germany [21]) to calculate oxygen uptake (VO_2_) and carbon dioxide production (VCO_2_). Calibration protocols were carried out according to the manufacturer’s instructions (before each experimental trial) to calibrate gas concentration and expired air volume. An average of VO_2_ and VCO_2_ for the last minute of each stage was used for statistical analysis [22]. Rates of energy expenditure and substrate oxidation (fat and carbohydrate) were calculated using the non-protein respiratory quotient [23]. In each trial, the maximal rate of fat oxidation MFO was individually calculated, and Fatmax was established as the percentage of VO_2_max at which MFO occurred.

Participants were asked to report the rating of perceived effort at the end of each stage during exercise using the Borg 6–20 points, as previously described [24]. In addition, side effects derived from *p*-synephrine intake were measured after exercise using a validated questionnaire [5]. This two phases survey has been effectively used to assess side effects resulting from acute *p*-synephrine ingestion in individuals performing several exercise situations [25].

### 2.5. Menstrual Cycle Phase

The regularity and duration of participants’ menstrual cycles were monitored for the previous 4 months before the beginning of the study through a mobile application (Mycalendar, Simpleinnovation, Susex, WI, USA) [26]. Participants completed a menstruation diary with this application that included: (i) menstrual duration, (ii) menstrual dates, and (iii) level of discomfort during the previous days leading up to the menstrual activity. Menstrual cycle duration was individually assessed, and the average was 28 ± 2 days (range from 25 to 30 days).

### 2.6. Statistical Analysis

Data are presented as means and standard deviation. Shapiro–Wilk tests were used to check the normality of all variables. Since all study outcomes were normally distributed, parametric tests were selected to examine differences between conditions. Two-way analysis of variance (ANOVA) was used to compare MFO, Fatmax, cardiovascular variables and tympanic temperature at rest, urine *p*-synephrine and 4-hydroxymandelic acid concentrations, and the magnitude of side effects derived from *p*-synephrine vs. placebo intake using a 2 × 2 model corresponding to time-of-the day × substance. A three-way ANOVA (2 × 2 × 6, corresponding to time-of-the day × substance × exercise intensity) was used to compare energy expenditure, fat, and carbohydrate oxidation rates, heart rate and the rating of perceived exertion during exercise. When a significant F value was obtained, a Bonferroni post hoc analysis was performed to determine pairwise differences for the values obtained with *p*-synephrine vs. placebo in the morning vs. evening for the same workload. Cohen’s formula for effect size (ES) was used for comparing differences between *p*-synephrine vs. placebo in morning and evening conditions, and the results were based on the following criteria: trivial (0–0.19), small (0.20–0.49), medium (0.50–0.79) and large (0.80 and greater). The significance level was set at *p* ≤ 0.050.

## 3. Results

Table 1 presents the descriptive parameters of the study’s participants. Six participants scored as morning type, nine as intermediate type, and one as evening type.

During the four experimental sessions, the air temperature was 21.3 ± 0.5 °C, and the relative humidity was 31 ± 4%, both controlled by a digital temperature and humidity monitor (Ambistik, Bad Kissingen, Germany).

There was no substance, time of day or substance × time of day effect on resting heart rate (F = 1.491–0.101; *p* = 0.241–0.755), and on systolic and diastolic blood pressure (F = 3.048–0.50; *p* = 0.101–0.827). However, there was a main effect of substance (F = 14.397; *p* = 0.002) and a main effect of time of day (F = 8.340; *p* = 0.011) on resting tympanic temperature, with no significant substance × time of day interaction (F = 0.203; *p* = 0.659). Compared to the placebo, *p*-synephrine intake increased the mean of tympanic temperature in the morning (36.08 ± 0.45 vs. 36.41 ± 0.40 °C, *p* = 0.033; ES = 0.52) and in the evening (36.41 ± 0.33 vs. 36.66 ± 0.25 °C, *p* = 0.018; ES = 0.56) while tympanic temperature was higher in the evening than in the morning for both placebo and *p*-synephrine trials.

**Figure 1:**
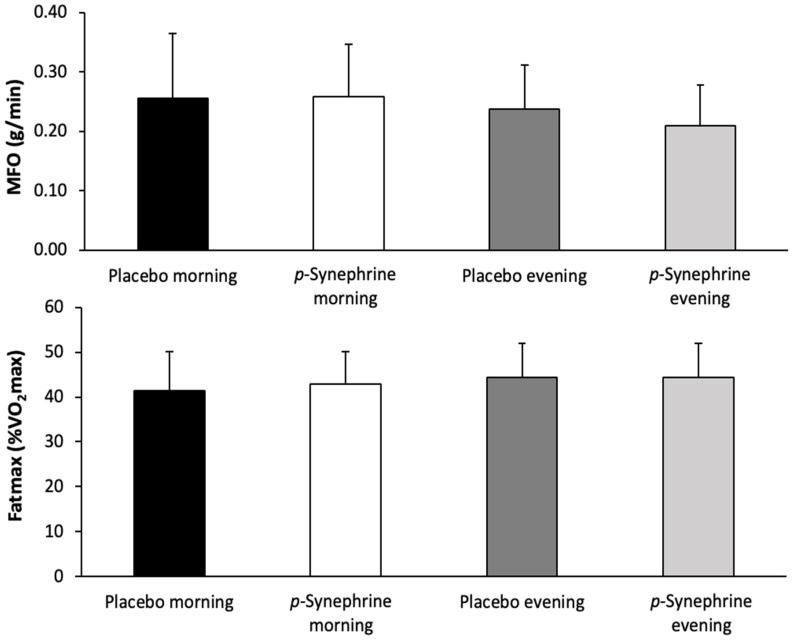
Maximal fat oxidation rate (MFO, in g/min; upper panel) and exercise intensity at MFO (Fatmax, as percentage of maximal oxygen uptake (VO_2_max); lower panel) during exercise of increasing intensity performed in the morning (8:00–10:00 h) or the evening (17:00–19:00 h) after the ingestion of 3 mg/kg of *p*-synephrine or a placebo in women.

Figure 1 depicts MFO and Fatmax in the morning and the evening, after the ingestion of *p*-synephrine or the placebo. There were no main effects of substance (F = 0.933; *p* = 0.349), time of day (F = 1.918; *p* = 0.186) and interaction on MFO (F = 0.874; *p* = 0.365). Similarly, there were no main effects of substance (F = 0.238; *p* = 0.633), time of day (F = 1.875; *p* = 0.191) or interaction (F = 0.319; *p* = 0.580) on Fatmax.

**Figure 2:**
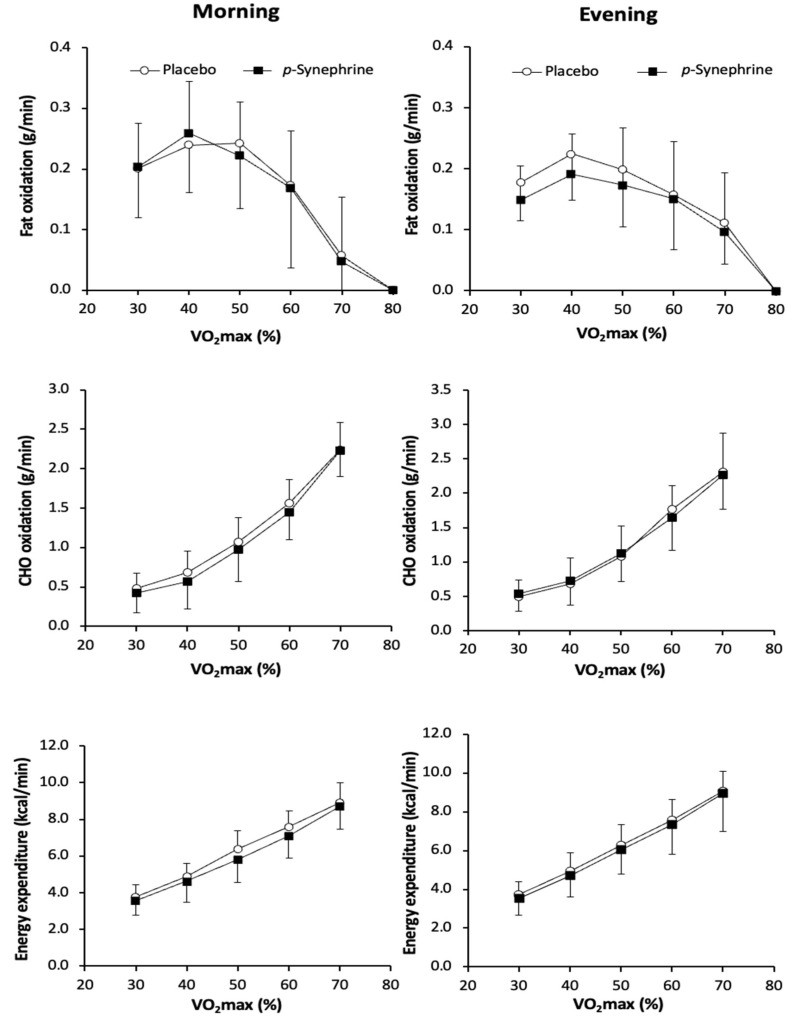
Rates of fat oxidation (in g/min), carbohydrate oxidation (in g/min) and energy expenditure (in kcal/min) during exercise of increasing intensity performed in the morning (8:00–10:00 h) or the evening (17:00–19:00 h) after the ingestion of 3 mg/kg of *p*-synephrine or a placebo in women.

Figure 2 presents data on the effect of *p*-synephrine intake on fat oxidation, carbohydrate oxidation and energy expenditure during the ramp exercise protocol in all experimental conditions. There were main effects of exercise intensity (F = 21.711; *p* < 0.001) and on the time of day (F = 17.000; *p* < 0.001) on the rate of fat oxidation during exercise. However, there was no main effect of substance on fat oxidation nor (F = 0.110; *p* = 0.745) three-factor interaction (F = 3.062; *p* = 0.060). Regarding carbohydrate oxidation rate during exercise, there was a significant main effect of exercise intensity (F = 98.268; *p* < 0.001), but there were no significant differences for substance (F = 0.518; *p* = 0.483), time of day (F = 0.591; *p* = 0.454) or interaction (F = 0.843; *p* = 0.546). There was a significant effect of exercise intensity (F = 164.841; *p* < 0.001) on energy expenditure rate, but with no effect of substance (F = 3.407; *p* = 0.085) and time of day (F = 3.563; *p* = 0.079). Additionally, there was a significant substance × time of day interaction on energy expenditure during exercise (F = 56.399; *p* < 0.001).

**Figure 3:**
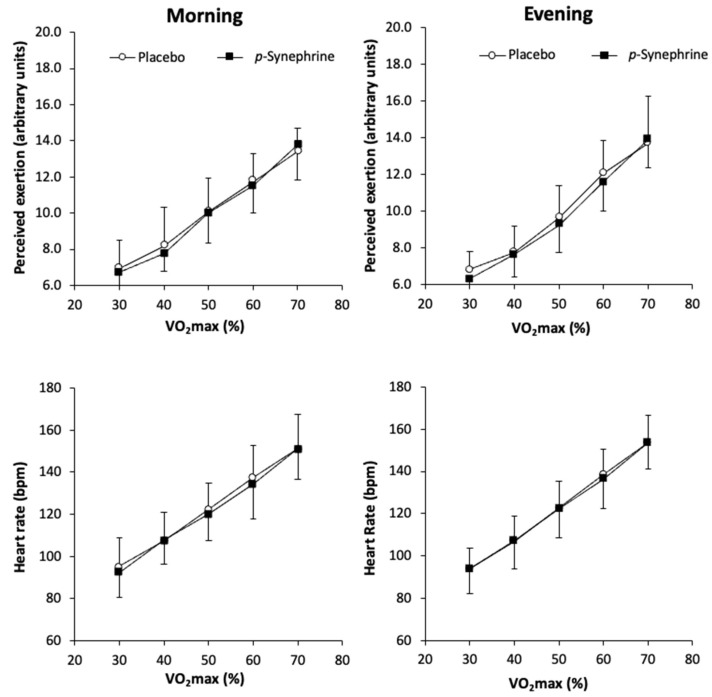
Rating of perceived exertion (in arbitrary units using the 6–20-point Borg scale) and heart rate (in beats per min; bpm) and during exercise of increasing intensity performed in the morning (8:00–10:00 h) or the evening (17:00–19:00 h) after the ingestion of 3 mg/kg of *p*-synephrine or a placebo in women.

Figure 3 depicts the effect of *p*-synephrine intake on the ratings of perceived exercise and heart rate during the ramp exercise protocol in all experimental conditions. There was a main effect of exercise intensity (F = 119.364; *p* < 0.001) on the rating of perceived exertion, but not a significant effect of substance (F = 0.932; *p* = 0.350), time of day (F = 1.220; *p* = 0.287) or interaction (F = 0.438; *p* = 0.779). There was a main effect of exercise intensity on heart rate (F = 112.520; *p* < 0.001) but not a significant effect of substance (F = 1.208; *p* = 0.289), time of day (F = 0.076; *p* = 0.787) or interaction on heart rate (F = 1.255; *p* = 0.340).

Table 2 depicts the magnitude of side effects after *p*-synephrine vs. placebo intake in the hours following exercise. There were no main effects of substance (F = 2.340–0.001; *p* = 0.152–0.966), time of day (F = 2.542–0.000; *p* = 0.137–1.000) or two-factor interaction (F = 1.370–0.409; *p* = 0.264–0.534) for the magnitude of the side effects reported by the participants. In all cases, the magnitude of the side effect was rated as lower than 3 a.u. by using a 1–10 a.u. scale.

There was a main effect of substance (F = 31.126; *p* < 0.001) on urine *p*-synephrine concentration with no effect of time of day (F = 1.150; *p* = 0.298) or substance × time of day interaction (F = 1.150; *p* = 0.298). Compared to the placebo, *p*-synephrine intake increased urine *p*-synephrine concentration in a similar magnitude in the morning (*p* < 0.001; ES = 1.95) and in the evening (*p* < 0.001; ES = 2.10, Table 3). Similarly, there was a main effect of substance (F = 5.998; *p* = 0.025) on urine 4-hydroxymandelic acid concentration with no effect of time of day (F = 1.496; *p* = 0.237) or substance × time of day interaction (F = 2.796; *p* = 0.113). Compared to the placebo, *p*-synephrine intake increased urine 4-hydroxymandelic acid concentration in the morning (*p* = 0.041; ES = 1.00) but not in the evening (*p* = 0.115; ES = 0.68).

## 4. Discussion

In this double-blind, placebo-controlled study, the outcomes indicate that a single dose of 3 milligrams of *p*-synephrine per kilogram of body mass did not significantly increase fat oxidation rate, neither in the morning nor in the evening, in physically active women. These results have important clinical implications since they suggest that *p*-synephrine may not be an effective substance to enhance the use of fat during exercise in women, drastically limiting its applicability for those women involved in exercise programs for weight loss/body fat reduction. The results of this study are different to previous investigations carried out in samples of men or mixed samples where women were only a minor portion of the sample, where *p*-synephrine in the same dosage and form of administration as the one used in the current investigation was effective in enhancing the rate of fat oxidation during exercise at 30–80% of VO_2_max [7,8,9,10,11]. However, the lack of effectiveness of *p*-synephrine to enhance fat oxidation in women is not novel, as it has been recently reported despite maintaining dose, form of administration and fitness level of participants [12]. Collectively, all this information suggests that *p*-synephrine may be a less effective substance that modifies substrate oxidation in women compared to men. Additionally, it seems that the time of day is not a factor explaining the lack of effectiveness of this substance to enhance fat oxidation during exercise in women.

The earlier investigation suggesting that *p*-synephrine does not improve fat oxidation during exercise in healthy and active women [12] also found that this substance produced an increase of 0.3 °C in resting tympanic temperature. These authors [12] argued that this higher basal body temperature, induced by the acute intake of *p*-synephrine, somewhat diminished the potential effect of *p*-synephrine on substrate oxidation, as higher body temperature reduces the utilization of fat during exercise [19]. The current investigation agrees with this previous finding, as the tympanic temperature was higher in both *p*-synephrine trials than in the placebo time-of-day-matched trials. Specifically, *p*-synephrine increased tympanic temperature by 0.33 °C in the morning and by 0.25 °C in the evening. Additionally, the changes in urine *p*-synephrine and 4-hydroxymandelic acid concentrations 60 min after *p*-synephrine intake were of similar magnitude in the morning and the evening (Table 3). These outcomes suggest that thermogenic responses and substance metabolism to acute *p*-synephrine intake are similar in the morning and the evening, at least in women. Additionally, the higher tympanic temperature with *p*-synephrine at both times of day, together with the lack of improvement in fat oxidation during a wide range of aerobic exercise intensities (Figure 1 and Figure 2), reinforces the hypothesis that higher body temperature is a potential interfering factor to obtaining *p*-synephrine-induced changes in substrate oxidation during exercise. As previous studies have found that physiological responses to acute *p*-synephrine intake are different in men and women [27,28], we assume that the sex-specific differences in response to *p*-synephrine intake during exercise is associated with its “thermogenic effect”, present only in women, rather than a diurnal variation.

The current findings offer additional scientific evidence versus those provided by previous research studies concerning MFO and Fatmax diurnal variation in male subjects [14,15]. Most studies of circadian rhythms to date have focused on males, observing that both MFO and Fatmax seem to be increased in the evening compared with the morning in active young men (+7%, and +12%, respectively) [13], normal-weight and obese men (+9%, and 11%, respectively), and male endurance-trained athletes (+15%, and +8%, respectively) [14,15]. However, a recent study has reported that MFO and Fatmax rates were similar in the morning and the evening in female subjects [16], although this is not always the case [29]. The current investigation confirms the lack of within-day fluctuations of MFO and Fatmax in active women, as these variables were unaffected when comparing the same exercise protocol performed in the morning (8–10 am) and evening (5–7 pm). The explanation for the lack of a circadian rhythm associated with fat oxidation during exercise in women is not evident from the data of this study. Previous works related to the diurnal variation of MFO and Fatmax have attributed the above-mentioned sex-specific differences to specific time-of-day-related physiological variables [30]. Concretely, core body temperature reaches its daily peak in the evening, leading to a subsequent increment of energy metabolism and enhancing actin–myosin cross-bridging and muscle compliance, all of them important factors in optimizing fat oxidation during endurance exercise [31]. Furthermore, it is also known that the catecholamine peak in response to exercise is considerably higher in the evening than in the morning [31,32,33]. Catecholamines significantly stimulate increased free fatty acids release and optimize lipolysis processes of both adipose and skeletal muscle tissues, therefore promoting higher rates of fat oxidation during exercise, especially in women, due to their higher percentage of body fat. In addition, women store more fat in the gluteal–femoral region, whereas men store more fat in the visceral (abdominal) depot [34]. Taken all together, these time-of-day physiological peculiarities in response to exercise may explain the diurnal variation of MFO and Fatmax in the above-mentioned studies, carried out in cohorts of men [13,14,15,29]. However, they do not explain why these variations do not induce higher fat oxidation rates in the evening in women, despite participants of the current study having higher resting tympanic temperatures in the evening. Hence, further investigation should be carried out comparing fat oxidation rates at different times of day in men and women within the same experimental protocol to determine a possible explanation for this sex-specific difference.

Most of the experiments in the field of exercise metabolism have been conducted on men, considering that skeletal muscle fiber architecture and enzymatic oxidative activity—among other parameters—are similar during exercise in both endurance-trained men and women [35,36]. This assumption represents an important sex bias since they found a superior contribution of fat metabolism during extensive endurance exercise in female athletes than in their male counterparts [37]. In this sense, it has been proposed that female sex hormones may play an important role in lipid metabolism during exercise, especially estrogen and progesterone [38,39]. Concretely, the study of D’Eon et al. [40] concluded that the exogenous administration of estradiol improved fat oxidation rates in response to 60 min of continuous aerobic exercise at moderate intensity in healthy women whose estrogens blood concentrations were previously reduced until postmenopausal levels. These increments were directly associated with greater free fatty acid concentrations in plasma [40], which are one of the most important factors to reach higher levels of MFO [41]. Given that plasma levels of estrogens are relatively elevated during the luteal phase—all female athletes performed exercise tests during this phase of the menstrual cycle—their hypothetical increment of free fatty acid blood concentration could be a sufficient stimulus to counteract the hypothetical diurnal variation of MFO and Fatmax. Thus, upcoming studies are requested to confirm whether the present findings apply to all phases of the menstrual cycle.

In the current experiment, we obtained data on heart rate and blood pressure at rest. Additionally, we questioned the participants about side effects either after the ingestion of the placebo or *p*-synephrine. The ingestion of 3 mg/kg of *p*-synephrine did not modify cardiovascular variables at rest in the morning or in the evening while the magnitude of side effects was minor and equivalent with placebo and *p*-synephrine intakes at both times of the day. This investigation provides further evidence to consider *p*-synephrine as a safe substance, at least for healthy individuals, coinciding with previous reports in both human and animal studies [5,42,43]. However, more investigations are needed in other populations of individuals that may use *p*-synephrine to enhance fat oxidation during exercise, as most of the reports indicating lack of pernicious effects after *p*-synephrine intake have been carried out in young and healthy individuals.

Despite the strengths and novelty of this investigation, this study has some limitations that should be recognized: (i) We only included young female and physically active participants, and therefore, no extrapolation can be assumed for other populations such as elite athletes, obese/overweight women, or older women. (ii) Although we organized all exercise trials during the luteal phase of the menstrual cycle, plasma levels of female sexual hormones (i.e., estrogen and progesterone) and free-fatty acid concentrations were not assessed; (iii) Body temperature and plasma catecholamines were not registered during the exercise test, and therefore, no confirmation about their role on the MFO and Fatmax diurnal variation in women can be offered. (iv) We used the “Fatmax” test with 3 min stages to assess both MFO and Fatmax within an exercise testing of 15 min (3 min × 5 stages) that entails low levels of fatigue. However, it has been previously suggested that the use of 3 min stages overestimates the assessment of fat oxidation (~0.02 g/min) while it underestimates carbohydrate oxidation (~0.07 g/min) in comparison to the use of 6 min stages [44]. Nevertheless, these effects minimally affected the outcomes of the study, as the potential overestimation/underestimation of fat/carbohydrate oxidation would be equally present in all four experimental trials. In any case, the lack of effect of *p*-synephrine intake in fat oxidation in women should be confirmed in more ecologically valid exercise protocols of prolonged and fixed-load exercise (e.g., 1 h at Fatmax), as previously conducted in men [11].

## 5. Conclusions

The main findings of the present study indicate that the acute intake of 3 mg/kg of *p*-synephrine before exercise did not increase MFO and Fatmax independently of the time of the day in women. The intake of *p*-synephrine did not modify the relationship between fat oxidation and exercise intensity in the morning or evening trials. This information suggests that *p*-synephrine is not an effective substance to modify substrate oxidation toward a higher reliance on fat during exercise in women, while the time of day is not a factor explaining the lack of effectiveness of this substance. Hence, the utilization of *p*-synephrine-containing products in exercise programs for weight loss/body fat reduction in women is not supported by the findings of this investigation. The *p*-synephrine-induced increase in tympanic temperature, found before exercise in both morning and evening trials, likely prevented the effect of *p*-synephrine on fat oxidation during exercise.

## Figures and Tables

**Table 1:** Participants’ age, morphological characteristics, and maximal values at the end of a VO_2_max test on a cycle ergometer.

Variable (Units)	Mean ± SD	Range
Age (yr)	26.7 ± 8.7	18.0–48.0
Body mass (kg)	61.6± 9.1	47.9–83.5
Body height (cm)	166.0 ± 7.0	152.0–175.0
Fat mass (%)	24.2 ± 5.8	15.0–39.2
VO_2_max (mL/kg/min)	39.4 ± 6.6	30.4–50.2
Maximal heart rate (beats/min)	179.0 ± 6.1	170–189
Maximal wattage in VO_2_max test (w)	207.3 ± 30.7	170–275

**Table 2:** Magnitude of side effects for the 24 h following the ingestion of 3 mg/kg of *p*-synephrine or a placebo in the morning (8:00–10:00 h) or evening (17:00–19:00 h) in women.

Variable (Units)	Placebo Morning	*p*-Synephrine Morning	Placebo Evening	*p*-Synephrine Evening
Nervousness (a.u.)	1.2 ± 0.6	1.6 ± 1.4	1.3 ± 0.9	1.5 ± 0.7
Vigor (a.u.)	1.4 ± 0.7	1.8 ± 2.3	2.1 ± 1.7	1.9 ± 1.7
Irritability (a.u.)	1.2 ± 0.6	1.8 ± 1.1	1.7 ± 1.7	1.2 ± 0.4
Muscle pain (a.u.)	1.3 ± 0.8	2.3 ± 1.5	2.1 ± 1.4	2.1 ± 1.8
Gastrointestinal distress (a.u.)	1.2 ± 0.6	1.7 ± 1.8	1.0 ± 0.3	1.2 ± 0.4
Diuresis (a.u.)	1.5 ± 0.8	1.3 ± 0.6	2.1 ± 2.1	1.6 ± 1.7
Insomnia (a.u.)	3.2 ± 2.7	2.1 ± 2.3	2.5 ± 2.9	2.6 ± 2.7

The magnitude of each side effect was self-reported by using a 1-10-arbitrary units (a.u.) scale. Participants were previously informed that one point meant a minimal amount of that item and 10 points meant a maximal amount.

**Table 3:** Urine *p*-synephrine and 4-hydroxymandelic acid concentrations after the ingestion of 3 mg/kg of *p*-synephrine or a placebo in the morning (8:00-10:00 h) or the evening (17:00-19:00 h) in women.

Variable (Units)	Placebo Morning	*p*-Synephrine Morning	Placebo Evening	*p*-Syneprhine Evening
Urine *p*-synephrine concentration (µg/L)	102.22 ± 22.23	23,531 ± 24,074	65.31 ± 25.99	17,106 ± 16,252
Urine 4-hydroxymandelic acid concentration (µg/L)	0.40 ± 0.01	3.03 ± 5.24	0.65 ± 0.31	1.31 ± 1.65

## Data Availability

Not applicable.
